# Supplementary material for: Decision level integration of unimodal and multimodal single cell data with scTriangulate
Source: Nat Commun. 2023 Jan 25;14:406. doi: 10.1038/s41467-023-36016-y (PMC9876931; doi:10.1038/s41467-023-36016-y)
Supplement: Supplementary file 1 — Supplementary Information [file 41467_2023_36016_MOESM1_ESM.pdf]

Supplementary Figure 1

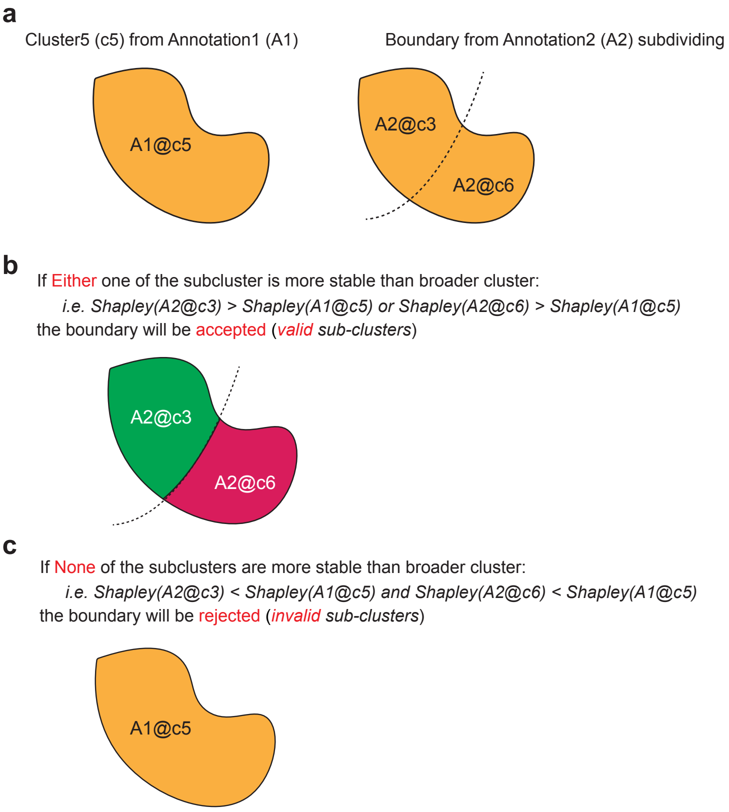

Supplementary Figure 1. Defining stable versus unstable subclusters with scTriangulate. A theoretical example is outlined for the determination of stable subclusters via comparison of the Shapley Value statistic. a) One cluster (c5) in annotation-set A1 (left) is divided by an imaginary boundary from an overlapping annotation-set (A2) (right). b) Under the condition where either one of the subclusters (A2@c3, or A2@c6) is more stable than the broader cluster (A1@c5), based on the Shapley Value derived from the reassign, TF-IDF, and SCCAF scores, this boundary will be kept and serve as a valid division that demarcates two stable subpopulations. c) Alternatively, under the condition where none of the subclusters is more stable than the broader cluster, the program will reject this boundary.

Supplementary Figure 2

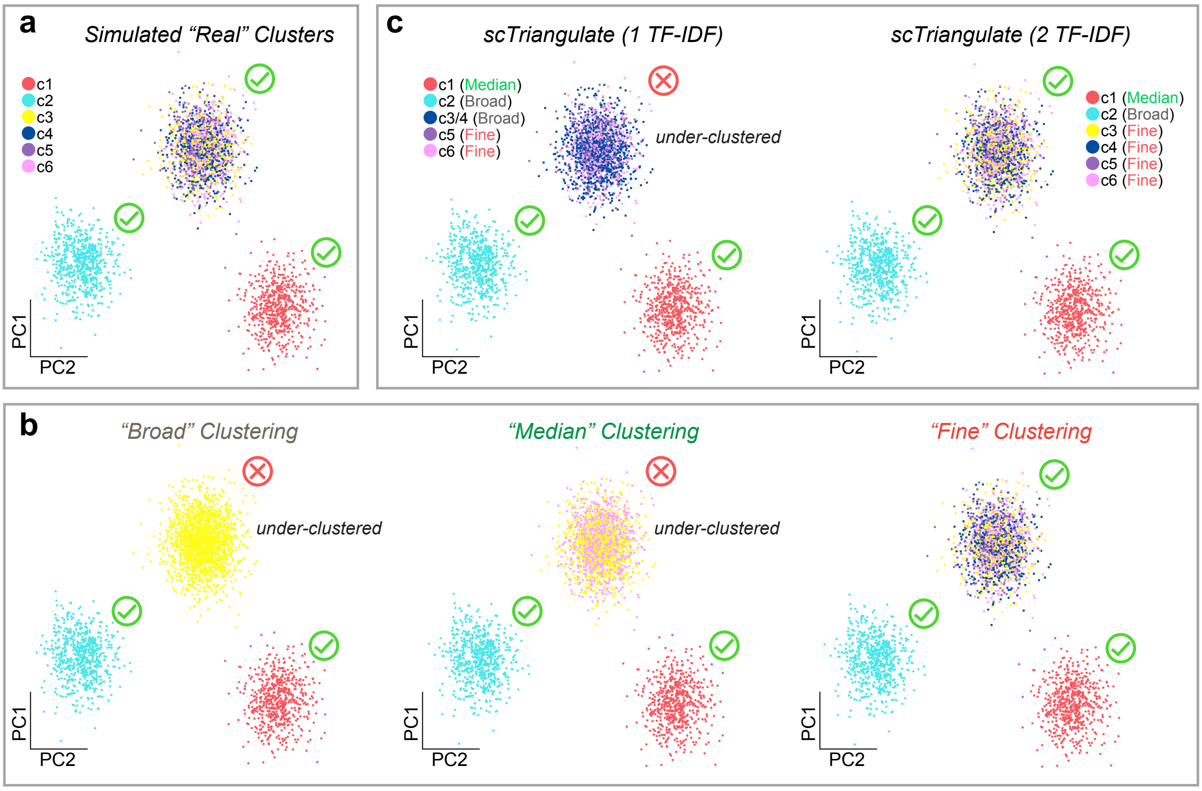

Supplementary Figure 2. Benefit of additional stability metrics in the triangulation. a) As a ground-state truth to assess the specificity of predictions by scTriangulate, we simulated scRNA-Seq data for two highly distinct simulated populations (c1 and c2) and four subtly different subclusters (c3, c4, c5, c6) (Methods). b) To predict such clusters, we produced three cluster annotations for the same cells in panel a, with different granular cluster definitions (broad, median and fine). c) scTriangulate integration of Broad, Median and Fine clusters, using the default two TF-IDF scores (TF-IDF10 and TF-IDF5) or one TF-IDF score alone (TF-IDF10).

Supplementary Figure 3

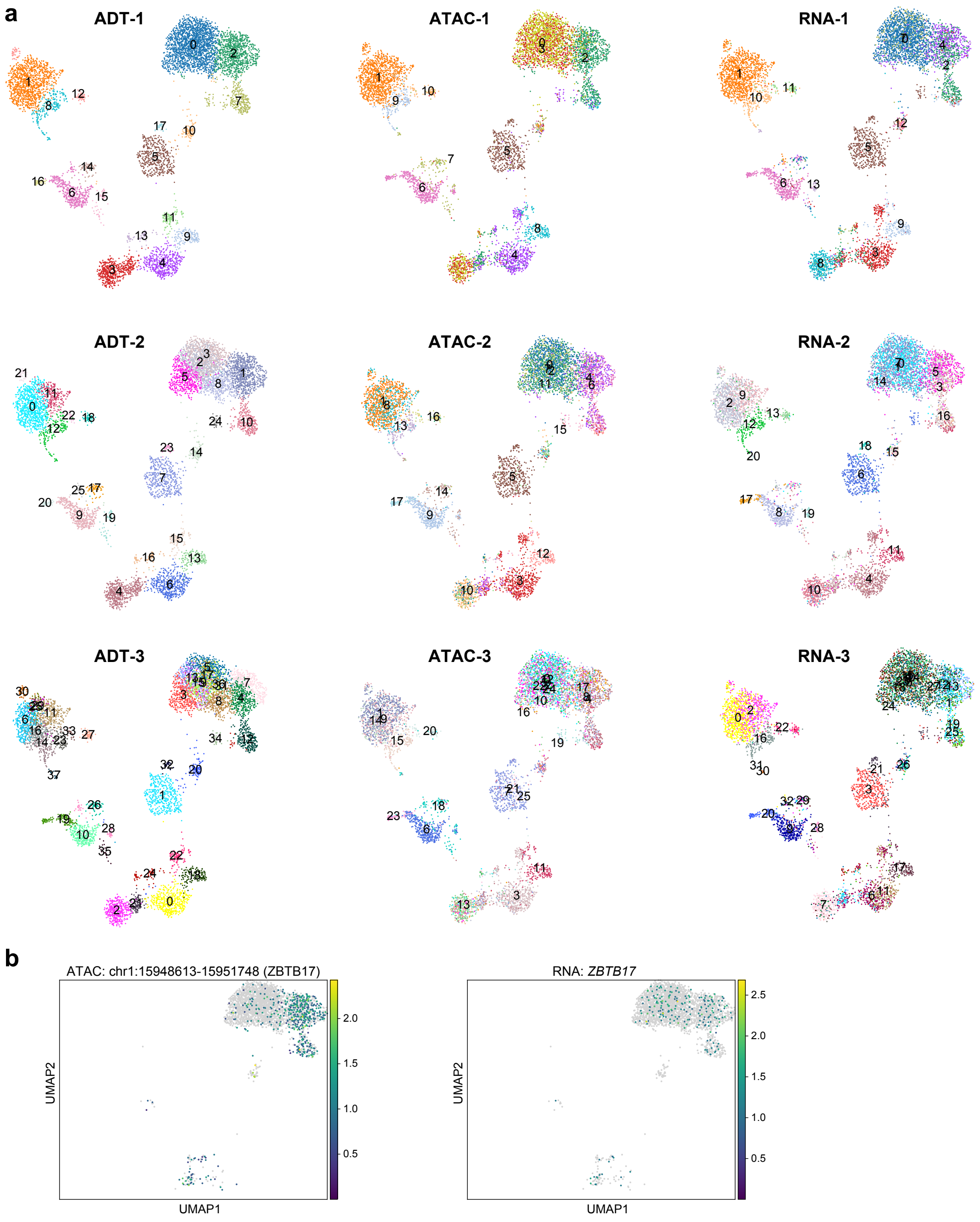

Supplementary Figure 3. Trimodal integration of PBMC clusters. a) Scanpy Leiden UMAP for multimodal analysis of human blood by TEA-Seq analysis (RNA+ATAC+ADT). UMAP coordinates are derived from the ADT data. Clustering results from three RNA, three ATAC and three ADT resolutions (r=1,2,3) are shown. b) Visualization of the top multiome ATAC marker (critical early T cell progenitor regulatory factor ZBTB17 18) for the CD95+ subset (left) and corresponding ZBTB17 gene expression in the same genomic loci and nuclei.

Supplementary Figure 4

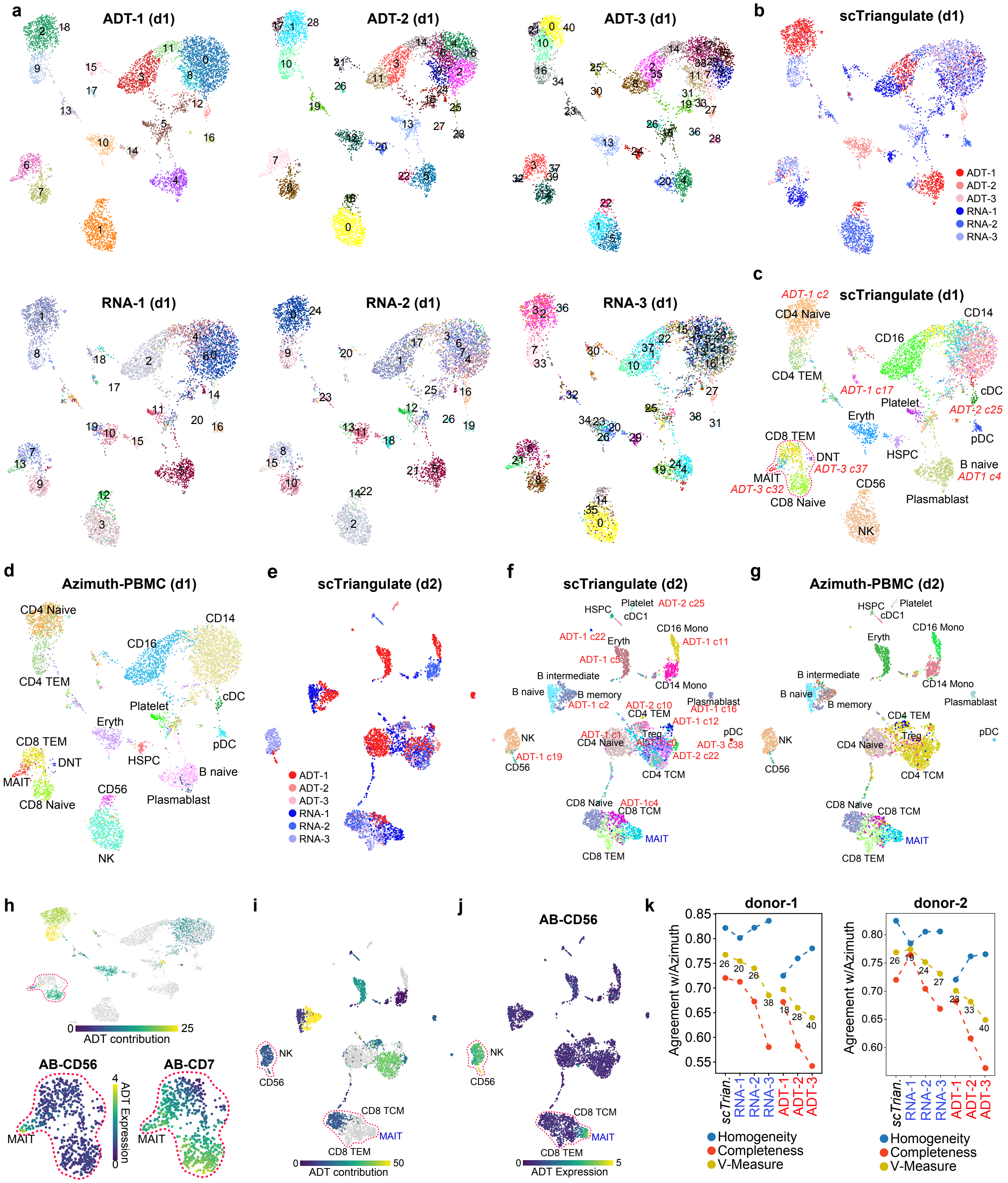

Supplementary Figure 4. Lymphoid subsets resolved through multimodal CITE-Seq integration. a) UMAP of CITE-Seq total nuclear cells (TNC) from donor 1, produced by scanpy Leiden clustering of three RNA and three ADT resolutions (r=1,2,3). b) scTriangulate final clusters with labels colored according to the source Leiden cluster and modality (donor 1). c) scTriangulate winning clusters after integration of multiple clustering resolutions (n=3) for each assayed modality relative to Azimuth annotations (donor 1). Clusters specifically derived from ADT cluster resolutions are labeled in the plot. d) Reference cell population labels from PBMC level-2 Azimuth projection (donor 1). e-g) Same as panels b,c and d, respectively, for CITE-Seq of donor 2. h) The selective contribution of ADTs is overlaid on the CITE-Seq UMAP (donor 1), based on the frequency of associated features among the top-20 markers of each final cluster (Methods). In the lower panel, the specificity of specific ADTs (CD56, CD7), demarcates the cluster boundaries of scTriangulate-defined MAIT cell subpopulations and CD8 T Effector Memory cell subpopulations, respectively. i) Relative contribution of ADT features to scTriangulate final clusters (donor 2). j) Independent evaluation of MAIT and NK CD56+ bright cell heterogeneity based on CD56 ADT expression (donor 2). k) Agreement with Azimuth PBMC reference label assignments measured by Homogeneity, Completeness, and V-Measure, for each individual or integrated set of clustering solutions (donor 1 left, donor 2 right). The number of clusters produced by each indicated resolution are displayed above the V-Measure data point. Source data are provided as a Source Data file.

Supplementary Figure 5

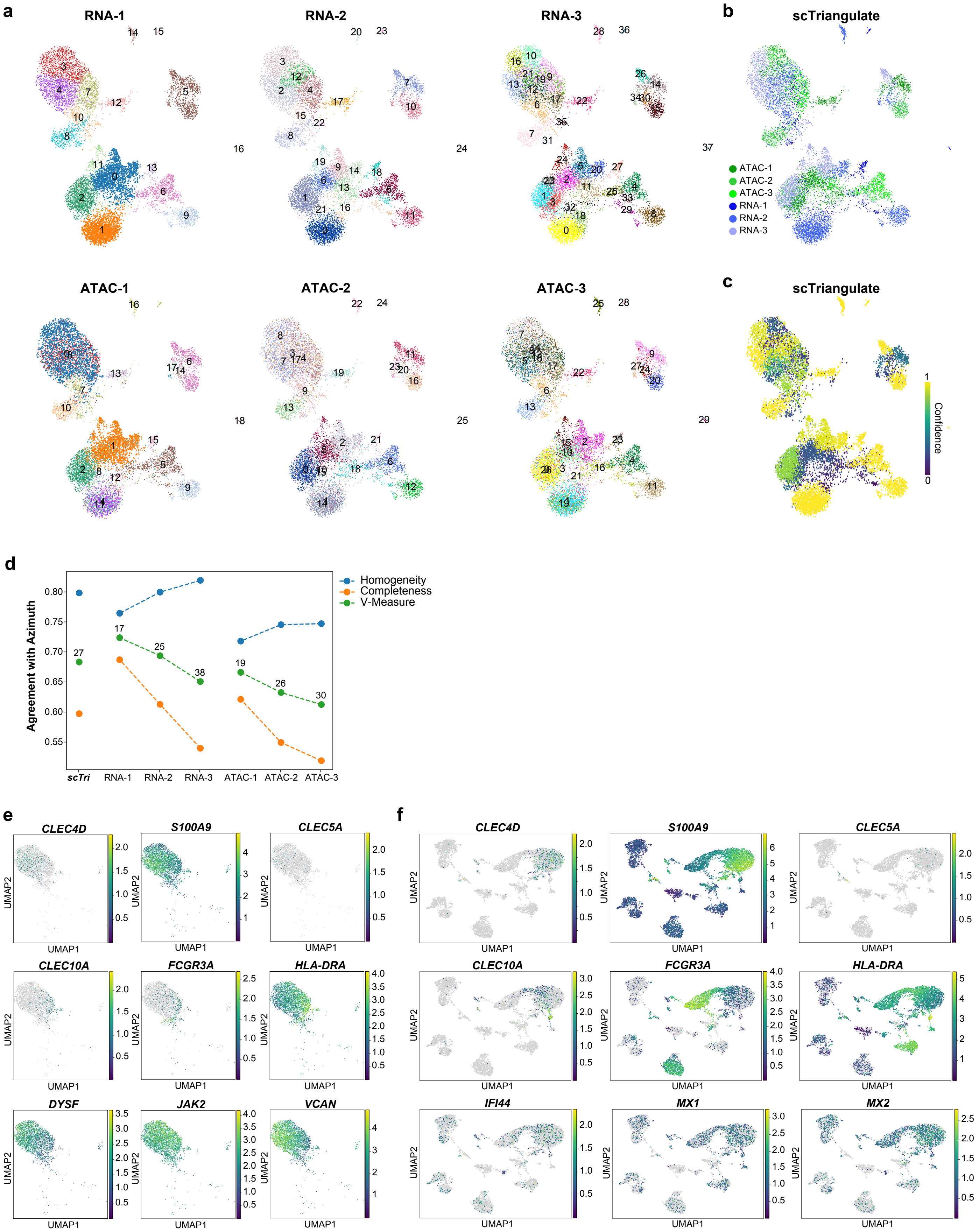

Supplementary Figure 5. CD14 monocyte subsets resolved through the integration of ATAC- and RNA. a) Scanpy Leiden UMAP for multimodal analysis of human blood by single-nuclei multiome analysis from three RNA and three ATAC resolutions (r=1,2,3). UMAP coordinates are derived from RNA. b) scTriangulate final clusters with labels colored according to the source Leiden cluster and modality. c) Cluster confidence for each final cluster (winning fraction, see Methods). d) Agreement with Azimuth PBMC reference label assignments measured by Homogeneity, Completeness, and V-Measure, for each individual or integrated set of multiome clustering solutions. e, f) Evidence for commonly detected subsets of monocytes in (e) multiome and (f) CITE-Seq with CD14 Monocytes. Canonical markers of monocyte heterogeneity with Azimuth defined CD14 Monocytes; classical CD14 Monocyte = S100A9, CLEC4D, CLEC5A; intermediate CD14 Monocyte = CLEC10A, FCGR3A, HLA-DRA; inflammatory monocyte = MX1, MX2, IFI44 and additional scTriangulate predicted top Monocyte population markers = DYSF, JAK2, VCAN. Source data are provided as a Source Data file.

Supplementary Figure 6

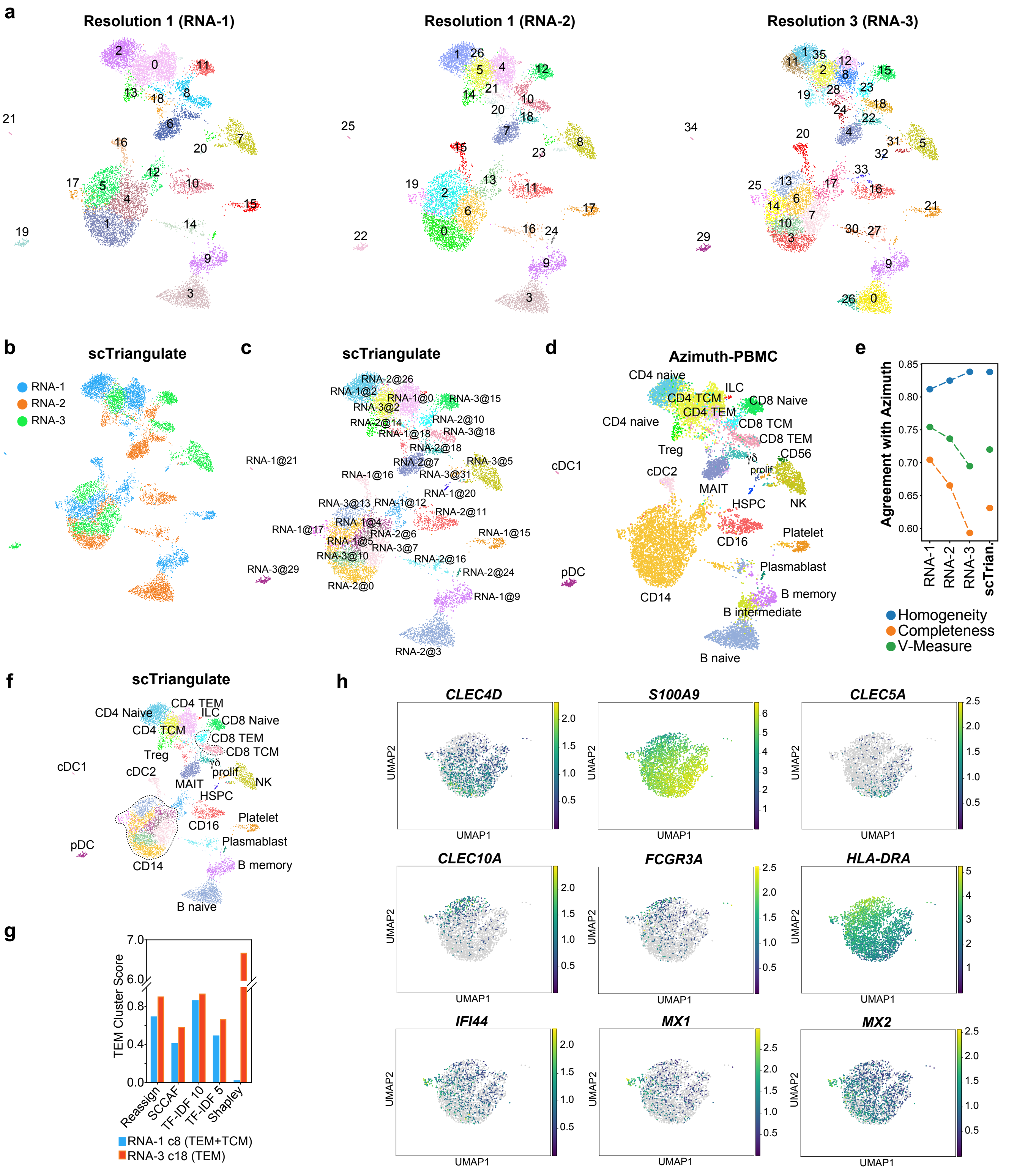

Supplementary Figure 6. Accurate prediction of PBMC scRNA-Seq consensus populations from different software resolutions. a) Scanpy Leiden clustering results for three resolutions (RNA-1, RNA-2, and RNA-3). b) scTriangulate final cluster source considering all evaluated resolutions. c) scTriangulate defined clusters after pruning and reclassification. The prefix of each label indicates the annotation source and the suffix the original cluster label from panel a. d) Projected labels from a PBMC CITE-Seq reference (Azimuth, Level 2). e) Agreement with Azimuth PBMC reference label assignments measured by Homogeneity, Completeness, and V-Measure, for each individual clustering solution. f) Overlaid cell-type annotations from Azimuth on scTriangulate results, with the delineation of rare CD8 T-cell subtypes highlighted in g and CD14 monocytes in h. g) Comparison of each stability metric and Shapley between a broader T cell memory cluster (light blue) and a more granular subset (T effector memory or TEM, red). h) Canonical markers of monocyte heterogeneity with Azimuth defined CD14 Monocytes; classical CD14 Monocyte = S100A9, CLEC4D, CLEC5A; intermediate CD14 Monocyte = CLEC10A, FCGR3A, HLA-DRA; and inflammatory monocyte = MX1, MX2, IFI44. Source data are provided as a Source Data file.

Supplementary Figure 7

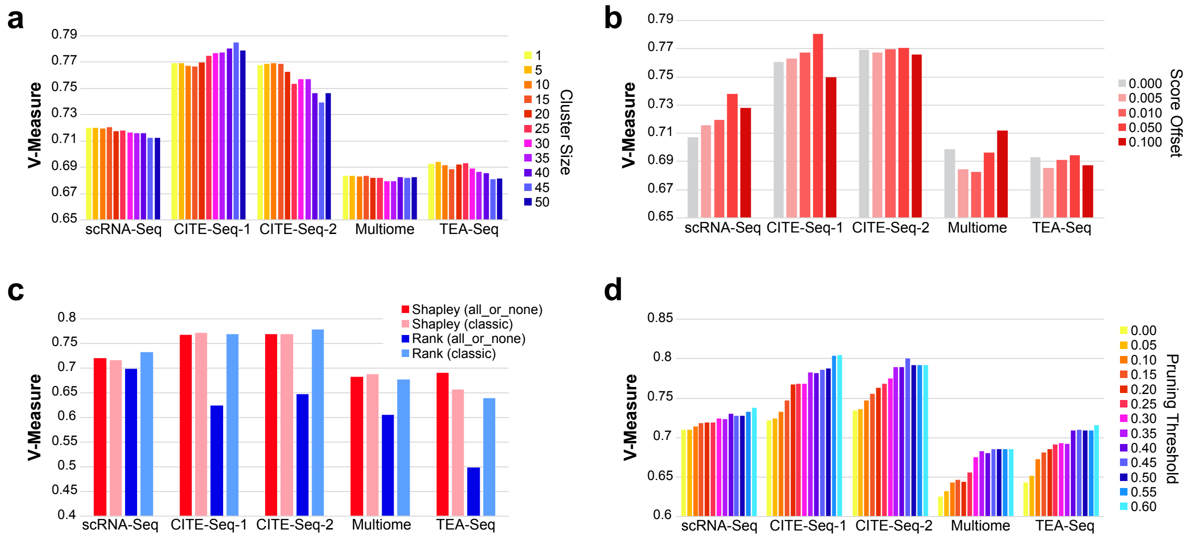

Supplementary Figure 7. Sensitivity analysis for distinct tunable parameters in scTriangulate. The performance of different scTriangulate tunable parameters were tested for the blood single-cell genomics datasets evaluated in this manuscript (unimodal and multimodal) (Methods). Each bar indicates the correspondence of clusters in scTriangulate when compared to the PBMC Azimuth reference (quantified by V-measure). a) Comparison of cutoffs for reliable minimum number of cells in a source cluster (Cluster Size) for all evaluated annotations, ranging from 1-50 cells. b) Comparison of different stability metric rank tolerance (offset) thresholds, ranging from 0.0-0.1. c) Comparison of different annotation importance strategies (Shapley and simple Rank-based), for different rank prioritization approaches (all_or_none, classical). All_or_none = winner takes all strategy. Classic = unadjusted rank strategy. d) Comparison different cluster pruning thresholds, ranging from 0-60% of cells retained from the original parent cluster. Source data are provided as a Source Data file.

Supplementary Figure 8

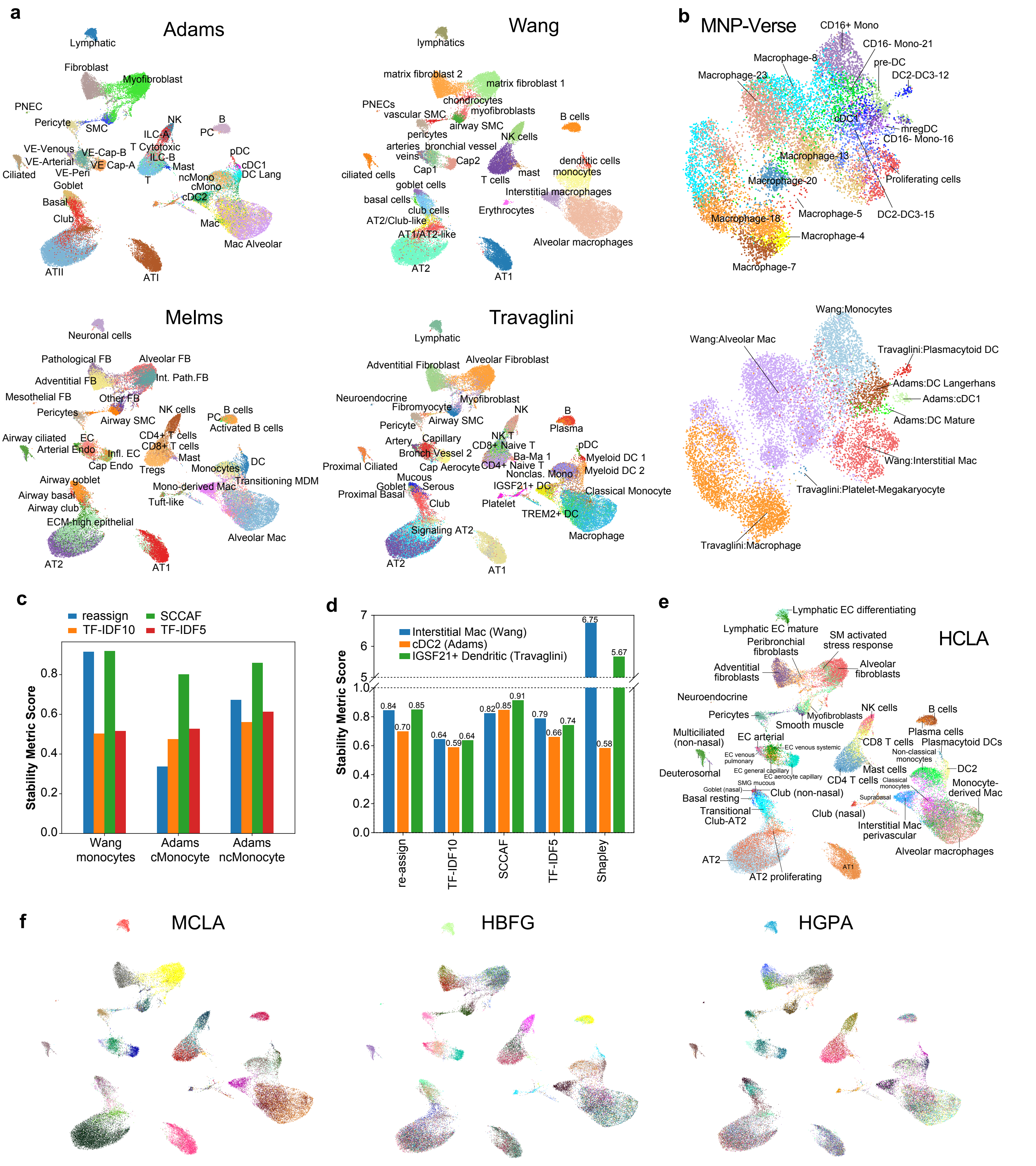

Supplementary Figure 8. Integrating multiple lung cell annotations to create a unified cell atlas. a) cellHarmony supervised mapping of scRNA-Seq reference labels (Melms et al. 2021 26, Adams et al. 2020 27, Travaglini et al. 2020 28) on to Wang et al. 2020 25 lung snRNA-Seq. b) UMAP of cell atlas annotations from MNP-Verse snRNA-Seq myeloid cell populations (top) and scTriangulate integrated predictions (bottom). c) Comparison of scTrianguate stability metrics for Wang monocytes, relative to Adams et al. 27 cMono and nMono. d) scTriangulate considered stability metrics for Wang Interstitial macrophages, compared specifically to overlapping Adams and Travaglini aligned cluster definitions. e) Cell annotations from the human lung cell atlas (HCLA) reference (Azimuth projected - finest level) onto the Wang snRNA-Seq data. f) Ensemble clustering of the supervised mapping assignments from panel a, using MCLA, HBGF and HGPA. Source data are provided as a Source Data file.

Supplementary Figure 9

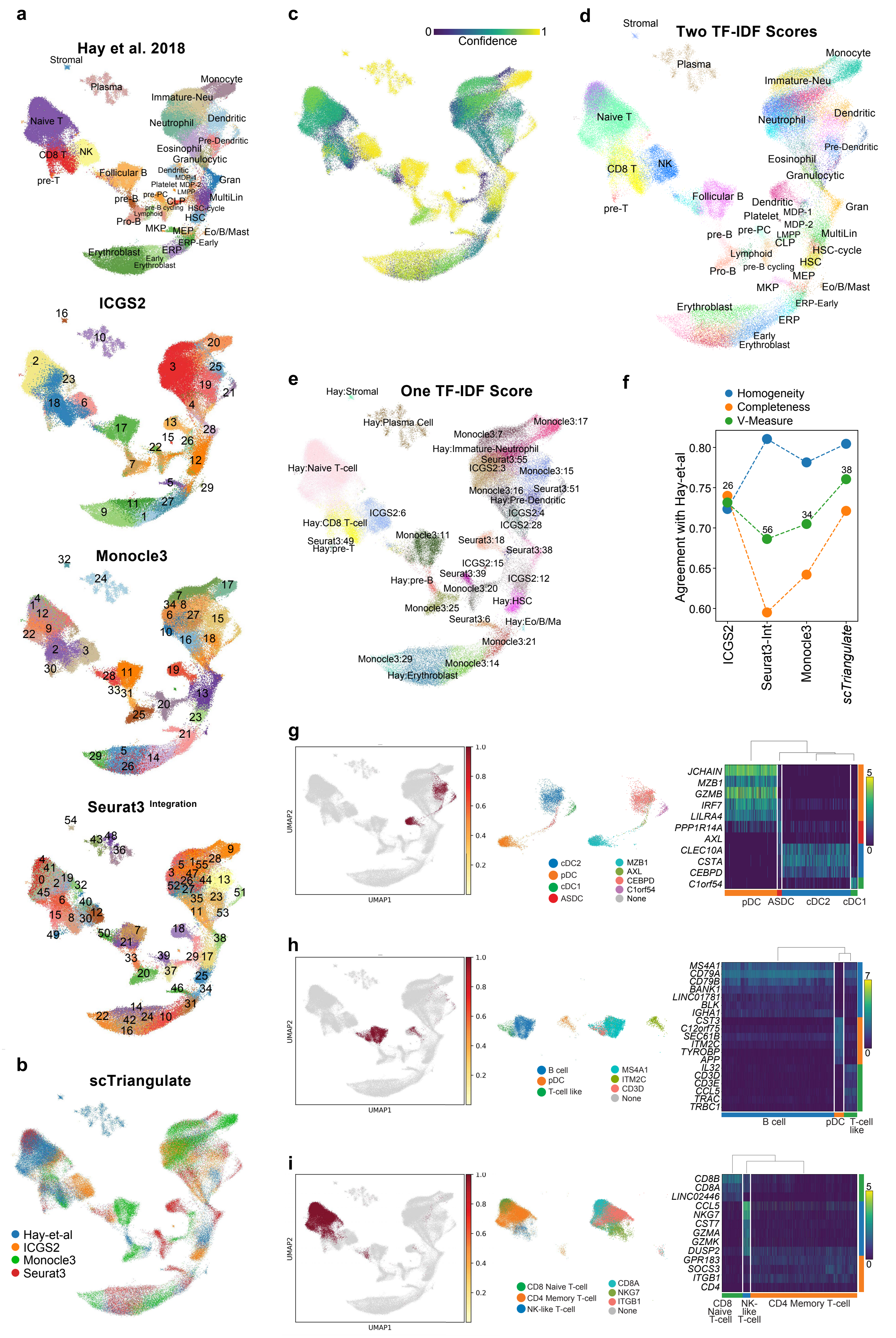

Supplementary Figure 9. Integration of diverse single-cell algorithms and curated annotations in Bone Marrow. a) UMAP of previously produced scRNA-Seq clustering results from three algorithms (ICGS2, Seurat3 and Monocle3) with the author source annotations (Hay et al. 2018) for an adult bone marrow atlas spanning >100,000 cells, 8 donors and 64 captures (10x Chromium 3' version 2). b) scTriangulate final cluster source from panel a. c) Cluster confidence for each final cluster (winning fraction, see Methods). d) scTriangulate results for the default setting parameters (two TF-IDF scores, TFIDF5 and TFIDF10), which are expected to produce more granular clusters (marker gene-driven Shapley Value). e) scTriangulate final results when only one TF-IDF score (TFIDF10) was applied (conservative). f) Agreement with Hay et al. cluster annotations for scTriangulate compared to Seurat 3 integration, Monocle 3 and ICGS2, measured by Homogeneity, Completeness, and V-Measure. g-i) The original author-identified (g) Dendritic Cell (DC) cluster, (h) Follicular B cells, and (i) Naive T cells (left), split into four subpopulations (middle) with support by canonical marker-gene expression (middle). Source data are provided as a Source Data file.

Supplementary Figure 10

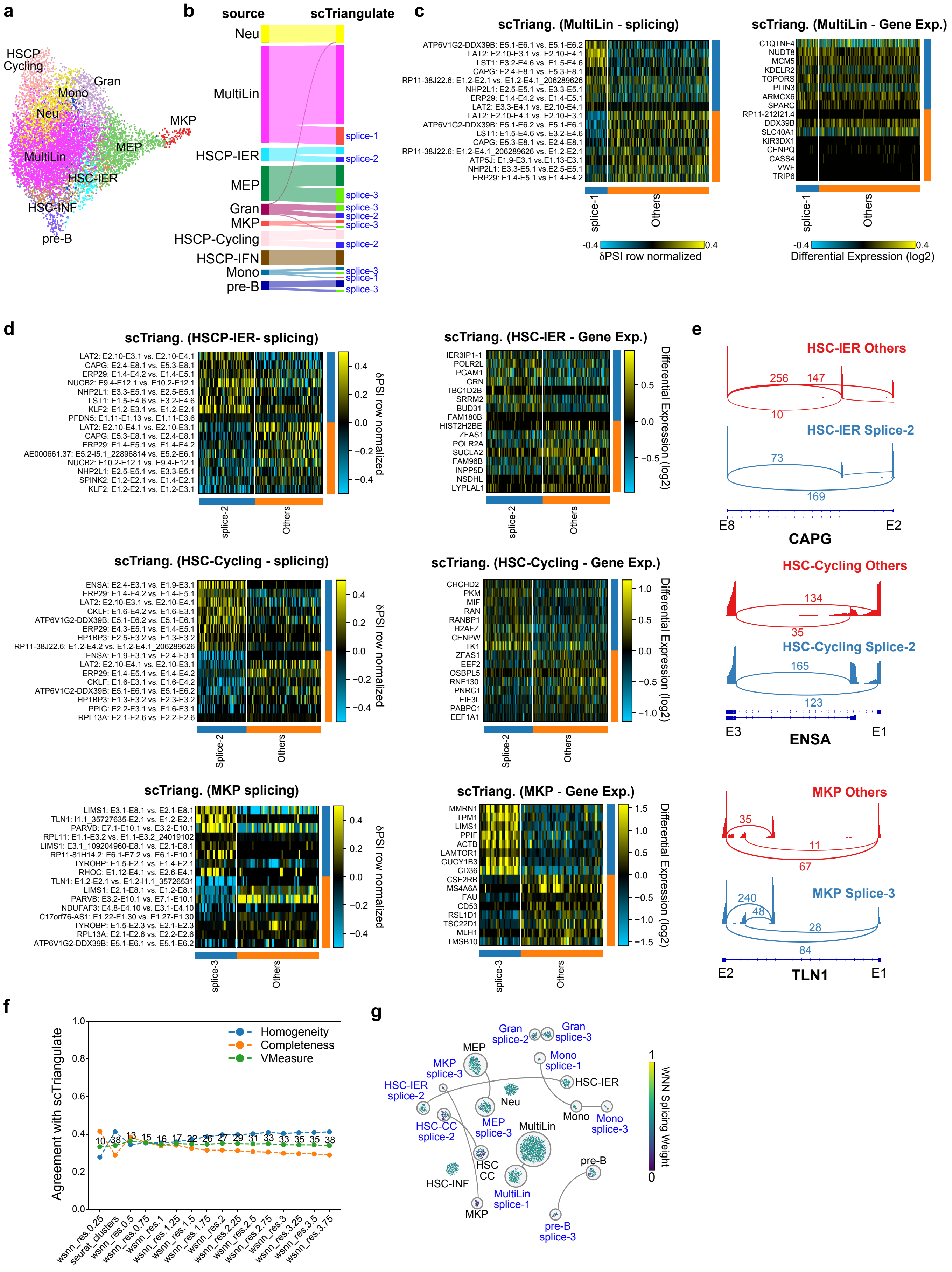

Supplementary Figure 10. Stable-splicing defined leukemic cell-population subtypes. a) UMAP of pediatric AML scRNA-Seq, produced from cellHarmony supervised cell assignments against normal CD34+ progenitor clusters. b) Sankey diagram of scTriangulate winning-cell populations from gene expression and splicing. Splicing-defined subclusters are denoted in blue along with the source splicing-NMF cluster number (1,2 or 3). c,d) Heatmaps displaying top discriminating splicing (left) and gene expression (right) markers that subdivide cellHarmony gene-expression defined cell populations. e) Representative SashimiPlots for marker splicing-events for scTriangulate winning clusters. f) Agreement with scTriangulate cluster annotations for a range of Seurat 3 WNN integration resolutions, measured by Homogeneity, Completeness, and V-Measure. The number of clusters produced by each indicated resolution are displayed above the V-Measure data point. g) Visualization of the Seurat WNN splicing "weight" on the scTriangulate AML UMAP. Source data are provided as a Source Data file.

Supplementary Figure 11

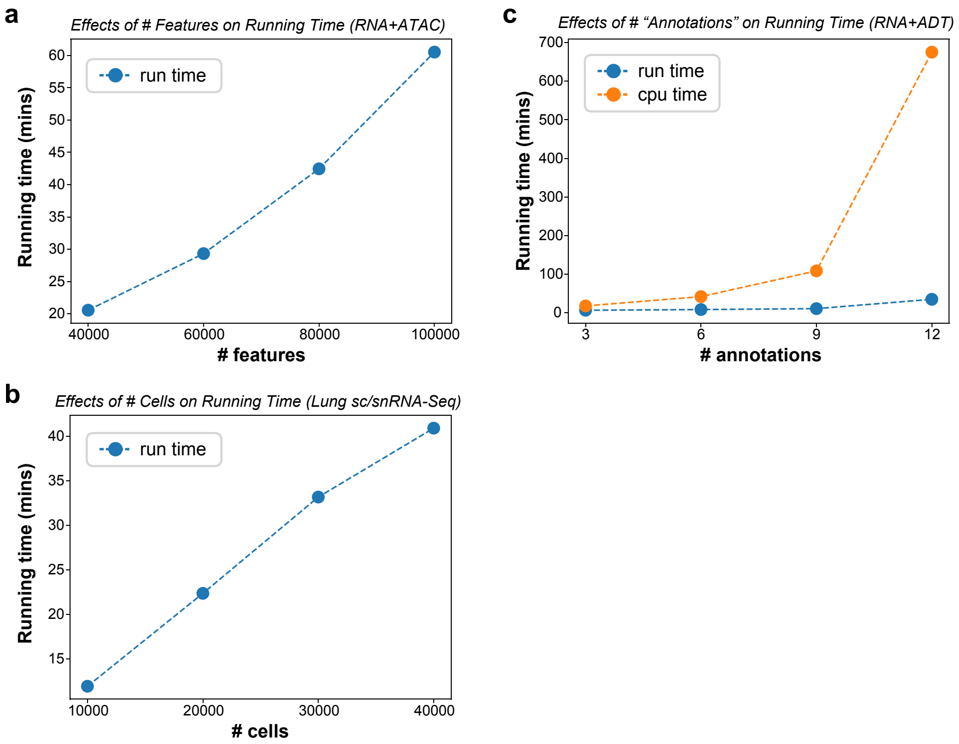

Supplementary Figure 11. Run time and scalability of scTriangulate. a) The impact of increasing numbers of features on runtime is shown for the multiome GEX+ATAC dataset (>100,000 features) in this study with randomly downsampling. b) The impact of increasing numbers of cells on runtime is shown for the Wang et al. Lung dataset (~46,000 single cells) 25 with all four annotation sources, with random downsampling of cell barcodes. c) The impact of increasing numbers of annotation-sets on runtime is shown for the CITE-Seq (sample 1) dataset (~6,000 single-cells), for 3, 6, 9, 12 query annotation-sets (in parallel, by default, blue line), compared with CPU time (sequentially, orange line). Source data are provided as a Source Data file.

Supplementary Table 1. CITE-Seq Antibody information

Antibody	BioLegend TotalSeq Cat #	Clone	Dilution
CD2	309229	TS1/8	1:200
CD3	300475	UCHT1	1:200
CD4	300563	RPA-T4	1:100
CD7	343123	CD7-6B7	1:200
CD8	301067	RPA-T8	1:200
CD10	312231	HI10a	1:100
CD11b	301353	ICRF44	1:100
CD13	301729	WM15	1:100
CD14	301855	M5E2	1:100
CD15	125615	MC-480	1:100
CD16	302061	3G8	1:100
CD19	302259	HIB19	1:100
CD20	302359	2H7	1:100
CD24	311137	ML5	1:100
CD33	366629	P67.6	1:200
CD34	343537	581	1:100
CD38	303541	HIT2	1:50
CD45	304064	HI30	1:100
CD45RA	304157	HI100	1:100
CD49f	313633	GoH3	1:200
CD56	392421	QA17A16	1:100
CD64	305037	10.1	1:200
CD66b	392905	6/40c	1:100
CD71	334123	CY1G4	1:200
CD90	328135	5E10	1:25
CD117	313241	104D2	1:100
CD123	306037	6H6	1:100
CD135(FLT3)	313317	BV10A4H2	1:100
CD235a	349117	HI264	1:200
HLADR	307659	L243	1:50
CD110 MPL	NA	S16017A	1:100
