# Supplementary material for: Decision level integration of unimodal and multimodal single cell data with scTriangulate
Source: Nat Commun. 2023 Jan 25;14:406. doi: 10.1038/s41467-023-36016-y (PMC9876931; doi:10.1038/s41467-023-36016-y)
Supplement: Supplementary file 3 — Reporting Summary [file 41467_2023_36016_MOESM3_ESM.pdf]

nature portfolio

Corresponding author(s): H. Leighton Grimes, Nathan Salomonis

Last updated by author(s): Dec 7, 2022

# Reporting Summary

Nature Portfolio wishes to improve the reproducibility of the work that we publish. This form provides structure for consistency and transparency in reporting. For further information on Nature Portfolio policies, see our Editorial Policies and the Editorial Policy Checklist.

## Statistics

For all statistical analyses, confirm that the following items are present in the figure legend, table legend, main text, or Methods section.

| n/a | Confirmed | |
|---|---|---|
| ☐ | ☒ | The exact sample size (*n*) for each experimental group/condition, given as a discrete number and unit of measurement |
| ☐ | ☒ | A statement on whether measurements were taken from distinct samples or whether the same sample was measured repeatedly |
| ☐ | ☒ | The statistical test(s) used AND whether they are one- or two-sided<br>*Only common tests should be described solely by name; describe more complex techniques in the Methods section.* |
| ☐ | ☒ | A description of all covariates tested |
| ☐ | ☒ | A description of any assumptions or corrections, such as tests of normality and adjustment for multiple comparisons |
| ☐ | ☒ | A full description of the statistical parameters including central tendency (e.g. means) or other basic estimates (e.g. regression coefficient) AND variation (e.g. standard deviation) or associated estimates of uncertainty (e.g. confidence intervals) |
| ☐ | ☒ | For null hypothesis testing, the test statistic (e.g. *F*, *t*, *r*) with confidence intervals, effect sizes, degrees of freedom and *P* value noted<br>*Give P values as exact values whenever suitable.* |
| ☐ | ☒ | For Bayesian analysis, information on the choice of priors and Markov chain Monte Carlo settings |
| ☐ | ☒ | For hierarchical and complex designs, identification of the appropriate level for tests and full reporting of outcomes |
| ☐ | ☒ | Estimates of effect sizes (e.g. Cohen's *d*, Pearson's *r*), indicating how they were calculated |

*Our web collection on statistics for biologists contains articles on many of the points above.*

## Software and code

Policy information about availability of computer code

| | |
|---|---|
| Data collection | [CITE-Seq Total Nucleated Cell] CellRanger 3.1.0<br>[scRNA-Seq Pediatric AML CD34+ Progenitor] CellRanger 2.1.1 |
| Data analysis | [scRNA-Seq] scanpy 1.7.2 and scTriangulate 0.9.0 and Azimuth (a module of Seurat 4.0)<br>[CITE-Seq, Multiome and TEA-Seq] scanpy 1.7.2 and scTriangulate 0.9.0 and Seurat 4.0 and ClusterEnsembles 1.0.0 and scvi-tools 0.14.3<br>[Lung Atlas] cellHarmony (a module in AltAnalyze 2.1.4) and scanpy 1.7.2 and scTriangulate 0.9.0 and ClusterEnsembles 1.0.0 and Azimuth (a module of Seurat 4.0)<br>[Bone Marrow] ICGS2 (a module in AltAnalyze 2.1.4), Multi-CCA (Seurat3) and Monocle 3 and scanpy 1.7.2 and scTriangulate 0.9.0<br>[single-cell AML splicing] ICGS2 (a module in AltAnalyze 2.1.4), MultiPath-PSI (a module in AltAnalyze 2.1.4) and scanpy 1.7.2 and scTriangulate 0.9.0 and Seurat 4.0<br>[single-cell MPN mutation] cellHarmony (a module in AltAnalyze 2.1.4) and scanpy 1.7.2 and scTriangulate 0.9.0<br>[simuation benchmark] Splatter 1.16.1<br>Other custom scripts referred in the manuscripts can be found in our official scTriangulate GitHub repository reproducible folder (https://github.com/frankligy/scTriangulate/tree/main/reproduce) |

For manuscripts utilizing custom algorithms or software that are central to the research but not yet described in published literature, software must be made available to editors and reviewers. We strongly encourage code deposition in a community repository (e.g. GitHub). See the Nature Portfolio guidelines for submitting code & software for further information.

## Data

Policy information about availability of data

All manuscripts must include a data availability statement. This statement should provide the following information, where applicable:

- Accession codes, unique identifiers, or web links for publicly available datasets
- A description of any restrictions on data availability
- For clinical datasets or third party data, please ensure that the statement adheres to our policy

The peripheral blood CITE-Seq CellRanger processed count matrix (h5), scripts, relevant outputs and metadata have been deposited in Synapse under the accession ID syn26320566 (https://www.synapse.org/#!Synapse:syn26320566). The scRNA-Seq of pediatric AML CD34+ progenitors CellRanger processed count matrix (h5), scripts, relevant outputs and metadata have been deposited in Synapse under the accession ID syn47980679 (https://www.synapse.org/#!Synapse:syn47980679). Bone Marrow atlas count matrix (mtx) has been deposited in Synapse under the accession ID syn26320732 (https://www.synapse.org/#!Synapse:syn26320732). The scRNA-Seq pre-processed count matrices and genotyping of CD34+ MPN progenitor were downloaded from GEO (https://www.ncbi.nlm.nih.gov/geo/query/acc.cgi?acc=GSE117825). The scRNA-Seq preprocessed sparse-matrix counts (h5) for PBMC was obtained from 10x Genomics website (https://support.10xgenomics.com/single-cell-gene-expression/datasets/3.0.0/pbmc_10k_v3) and has been deposited to Synapse under the accession ID syn26320659 (https://www.synapse.org/#!Synapse:syn26320659). The PBMC Multiome pre-processed sparse matrix counts (h5) was downloaded from 10x Genomic website (https://www.10xgenomics.com/resources/datasets/pbmc-from-a-healthy835donor-granulocytes-removed-through-cell-sorting-10-k-1-standard-2-0-0) and has been deposited to Synapse under the accession ID syn26320419 (https://www.synapse.org/#!Synapse:syn26320419). The PBMC TEA-Seq data (RNA-ATAC combined h5 file) and ADT count (csv file) were downloaded from GEO (https://www.ncbi.nlm.nih.gov/geo/query/acc.cgi?acc=GSM4949911). Lung Cell Atlas scRNA-Seq and snRNA-Seq counts data and original cluster annotations were obtained from four independent studies (https://www.ncbi.nlm.nih.gov/geo/query/acc.cgi?acc=GSE161383, https://www.ncbi.nlm.nih.gov/geo/query/acc.cgi?acc=GSE171524, https://www.ncbi.nlm.nih.gov/geo/query/acc.cgi?acc=GSE136831, https://ega-archive.org/studies/EGAS00001004344). The MNP-Verse reference (.RDS file) was downloaded from author-provided weblink (https://gustaveroussy.github.io/FG-Lab/). All other relevant data supporting the key findings of this study are available within the article and its Supplementary Information files or from the corresponding author upon reasonable request. Source data are provided with this paper.

## Human research participants

Policy information about studies involving human research participants and Sex and Gender in Research.

| | |
|---|---|
| Reporting on sex and gender | Analyses carried out in this study are for individual patients/donors and as such do not make claims or inference regarding population-level observations. Hence, since no case versus control evaluations are performed, sex is not considered as a biological variable. Sex and approximate age range is reported for samples analyzed by RNA-Sequencing profiled in this study, in compliance with the Cincinnati Children's Hospital Medical Center (CCHMC) Institutional Review Board (IRB) studies (#2008-1186 and #2018-1602) |
| Population characteristics | For CITE-Seq analyses, de-identified human granulocyte colony-stimulating factor (G-CSF) mobilized total nucleated cells (TNCs) were obtained through apheresis from Cell Processing Core (CPC) at Cincinnati Children's Hospital Medical Center (CCHMC). Two young adult donors were separately profiled. Donor 1 (ND19-341) was obtained from a 28 year-old white male and donor 2 (ND19-446) from a 31 year-old white female. CD34+ positive flow cytometry sorted progenitors were isolated from whole blood from a pediatric AML patient (age > 1 and <18 years old) resistant chemotherapy at the time of diagnosis (donor ID = CF001, sample ID = CF001CD34D0). |
| Recruitment | For CITE-Seq, recruitment was through the Cell Processing Core (CPC) at Cincinnati Children's Hospital Medical Center (CCHMC). The AML patient or guardians were required to sign an informed consent document before participation. |
| Ethics oversight | This project uses biomaterials from healthy donors who have been consented under the Children's Hospital Medical Center IRB-approved (#2008-1186) Normal Donor Repository. The AML patient sample collection and analysis required approval from the IRB (#2018-1602). CCHMC follow all the rules and regulations pertinent to human subject protection. |

Note that full information on the approval of the study protocol must also be provided in the manuscript.

# Field-specific reporting

Please select the one below that is the best fit for your research. If you are not sure, read the appropriate sections before making your selection.

☒ Life sciences ☐ Behavioural & social sciences ☐ Ecological, evolutionary & environmental sciences

For a reference copy of the document with all sections, see nature.com/documents/nr-reporting-summary-flat.pdf

# Life sciences study design

All studies must disclose on these points even when the disclosure is negative.

| | |
|---|---|
| Sample size | n=2 donors for CITE-Seq. AML and MPN multimodal scRNA-Seq analyses are conducted for a single patient. Analyses carried out in this study are for individual patients/donors and as such do not make claims or inference regarding population-level observations. Hence, since no case versus control evaluations are performed, no sample-size calculation was performed. |

| | |
|---|---|
| Data exclusions | None noted |
| Replication | For CITE-Seq validation, two independent human Total Nucleated Cells (TNC) captures were used the observations are reproducible across two biological replicates. Other evaluations are for individual patients/donors and as such do not make claims or inference regarding population-level observations. |
| Randomization | Not relevant, see explanation in sample size section |
| Blinding | Not relevant, see explanation in sample size section |

# Reporting for specific materials, systems and methods

We require information from authors about some types of materials, experimental systems and methods used in many studies. Here, indicate whether each material, system or method listed is relevant to your study. If you are not sure if a list item applies to your research, read the appropriate section before selecting a response.

## Materials & experimental systems

| n/a | Involved in the study | |
|---|---|---|
| ☐ | ☒ | Antibodies |
| ☒ | ☐ | Eukaryotic cell lines |
| ☒ | ☐ | Palaeontology and archaeology |
| ☒ | ☐ | Animals and other organisms |
| ☒ | ☐ | Clinical data |
| ☒ | ☐ | Dual use research of concern |

## Methods

| n/a | Involved in the study | |
|---|---|---|
| ☒ | ☐ | ChIP-seq |
| ☒ | ☐ | Flow cytometry |
| ☒ | ☐ | MRI-based neuroimaging |

## Antibodies

Antibodies used

Antibody, BioLegend TotalSeq Cat #, Clone, dilution
CD2 309229 TS1/8 1:200
CD3 300475 UCHT1 1:200
CD4 300563 RPA-T4 1:100
CD7 343123 CD7-6B7 1:200
CD8 301067 RPA-T8 1:200
CD10 312231 HI10a 1:100
CD11b 301353 ICRF44 1:100
CD13 301729 WM15 1:100
CD14 301855 M5E2 1:100
CD15 125615 MC-480 1:100
CD16 302061 3G8 1:100
CD19 302259 HIB19 1:100
CD20 302359 2H7 1:100
CD24 311137 ML5 1:100
CD33 366629 P67.6 1:200
CD34 343537 581 1:100
CD38 303541 HIT2 1:50
CD45 304064 HI30 1:100
CD45RA 304157 HI100 1:100
CD49f 313633 GoH3 1:200
CD56 392421 QA17A16 1:100
CD64 305037 10.1 1:200
CD66b 392905 6/40c 1:100
CD71 334123 CY1G4 1:200
CD90 328135 5E10 1:25
CD117 313241 104D2 1:100
CD123 306037 6H6 1:100
CD135(FLT3) 313317 BV10A4H2 1:100
CD235a 349117 HI264 1:200
HLADR 307659 L243 1:50
CD110 MPL XXXXXX S16017A 1:100

Validation

Human total nucleated cells (TNCs) and CD34+ cells were isolated via AutoMacs Pro (Miltenyi) from G-CSF mobilized healthy donors' peripheral blood. All antibodies were titrated on both TNCs and CD34+ cells at 5 serially diluted concentrations (1:25, 1:50, 1:100, 1:200, 1:400). Staining index was calculated to guide selection of the appropriate amount of antibodies to use.
